# Knowledge mapping visualization analysis of the military health and medicine papers published in the web of science over the past 10 years

**DOI:** 10.1186/s40779-017-0131-8

**Published:** 2017-07-12

**Authors:** Xuan-ming Zhang, Xuan Zhang, Xu Luo, Hai-tao Guo, Li-qun Zhang, Ji-wei Guo

**Affiliations:** 1 Southwest Hospital, Third Military Medical University, Chongqing, 400038 China; 2 Department of Clinical Laboratory, Xinqiao Hospital, Third Military Medical University, Chongqing, 400037 China

**Keywords:** Military health, Military medicine, Knowledge mapping, CiteSpace

## Abstract

**Background:**

Military medicine is a research field that seeks to solve the medical problems that occur in modern war conditions based on public medicine theory.

**Methods:**

We explore the main research topics of military health and medical research in the web of science™ core collection (WoSCC) from 2007 to 2016, and the goal of this work is to serve as a reference for orientation and development in military health and medicine. Based on CiteSpace III, a reference co-citation analysis is performed for 7921 papers published in the WoSCC from 2007 to 2016. In addition, a cluster analysis of research topics is performed with a comprehensive analysis of high-yield authors, outstanding research institutions and their cooperative networks.

**Results:**

Currently, the research topics in military health and medicine mainly focus on the following seven aspects: mental health diagnoses and interventions, an army study to assess risk and resilience in service members (STARRS), large-scale military action, brain science, veterans, soldier parents and children of wartime, and wound infection. We also observed that the annual publication rate increased with time. Wessely S, Greenberg N, Fear NT, Smith TC, Smith B, Jones N, Ryan MAK, Boyko EJ, Hull L, and Rona RJ were the top 10 authors in military health and medicine research. The top 10 institutes were the Uniformed Services University of the Health Sciences, the United States Army, the United States Navy, Kings College London, Walter Reed National Military Medical Center, Boston University, Walter Reed Army Institute of Research, Walter Reed Army Medical Center, Naval Health Research Center, and the VA Boston Healthcare System.

**Conclusions:**

We are able to perform a comprehensive analysis of studies in military health and medicine research and summarize the current research climate and the developmental trends in the WoSCC. However, further studies and collaborations are needed worldwide. Overall, our findings provide valuable information and new perspectives and shape future research directions for further research in the area of military health and medicine.

## Background

Military health and medicine is an important field in biological and medical sciences. Military medicine plays a key role in various aspects, such as supporting and maintaining health, preventing injuries and diseases among military staff and enhancing the well-being of the military armed forces during war. In addition, military activities also involve other actions such as emergency public health crises, natural disasters, emerging conflicts and anti-terrorist campaigns during peacetime [1]. As a system of medical science, the purpose of military health and medicine is to protect the physical and mental health of military members and related personnel, prevent and treat bodily injuries and psychological damage, and enhance the combat effectiveness of military forces that conduct military activities during both war and peace [2–4]. Bibliometric analyses have been widely used in various areas to explore research topics, estimate the productivity of authors and institutions, and identify international cooperative networks in specific fields. Although many papers have investigated military health and medicine, reports are still lacking on the trends in military health and medicine publications. Therefore, a summary and analysis of the published papers are very significant to scholars who wish to gain expertise in research trends and keep abreast of cutting-edge findings [5]. In this study, we utilized CiteSpace III, a freely available Java application for visualizing and analyzing trends and patterns in scientific literature and is designed as a tool for progressive knowledge domain visualization, to analyze research articles published in the WoSCC from 2007 to 2016. The results may provide great reference value for the future development of military health and medicine.

## Methods

### Research objectives

In this study, we propose that the main research topics are the developmental rules of military health and medicine and that these rules can guide future research. The search terms “health”, “medicine” and “military” were used to create the following search queries: (topic = military health) OR (topic = military medicine). We chose all document types of papers for a search that was performed in the WoSCC database and automatically removed repeated papers using CiteSpace III. The timeframe was set to between 2007 and 2016, and a total of 7921 research papers in military health and medicine were included in the analysis. The data obtained by this retrieval strategy include military health and medical articles. However, the articles published at military research institutions and non-military research institutions cannot be distinguished.

### Data source and retrieval

With the help of the CiteSpace III software developed by Dr. Chen Chaomei, which is based on the JAVA platform, a diversified visualization analysis was performed on the retrieved military medicine literature. CiteSpace III uses annual rings to represent the citation frequency of the object in different time periods [6]. The research contents include a co-citation analysis, co-citation cluster analysis, high-yield authors, outstanding research institutions, and their cooperative networks of the military health and medicine study. Therefore, important information such as prominent military health and medicine disciplines, core research directions and outstanding research teams can be obtained.

### Search strategy

The search terms “health”, “medicine” and “military” were used to create the following search queries: (topic = military health) OR (topic = military medicine). All document types of military health and medicine were found in the WoSCC database, including research articles, review articles, meeting abstracts, and proceedings etc. Meanwhile, we removed duplicate papers using the “Remove duplicates” function of CiteSpace III. The timeframe was set to between 2007 and 2016, and a total of 7921 research papers in military health and medicine were included in the analysis. The data obtained by this retrieval strategy include military health and medicine articles. However, the articles published at military research institutions cannot be distinguished from those published at non-military research institutions.

## Results

### The number of articles published in military medicine in the past 10 years

Using the above retrieval strategy, we searched the WoSCC database for articles that matched the criteria and analyzed the retrieval results with the help of the result analysis function that comes with the WoSCC. The analysis of the trend in publication quantity in military health and medicine from 2007 to 2016 demonstrated that the number of publications from 2007 to 2016 was 424 (5.353%), 496 (6.262%), 512 (6.464%), 646 (8.156%), 801 (10.112%), 825 (10.415%), 881 (11.122%), 952 (12.019%), 1134 (14.316%), and 1250 (15.781%) per year.

### Reference co-citation analysis

The analysis and interpretation of technical literature network mapping is the core approach of literature information mining. In a literature network mapping, nodes represent the analysis objects, annual rings represent citation frequency and the color spectrum reflects the time range of the citations. The larger the node, the more times that an object had been cited during the time span, the thicker the single ring, the larger the number of citations in the given time slice. The connection line between two nodes indicates the co-cited relationships between two objects. The color of the connection line indicates the time of the first co-citation. The length and thickness of the connection line indicate the intensity of the connection between the two nodes. Some nodes are surrounded by a purple circle, indicating that extensive links exist between this node and the nodes in other fields. These nodes are often the hub of the discipline or knowledge domain and serve as a bridge for knowledge flow, which is of special significance in the node network and needs to be highlighted and analyzed [6, 7].

**Fig. 1:**
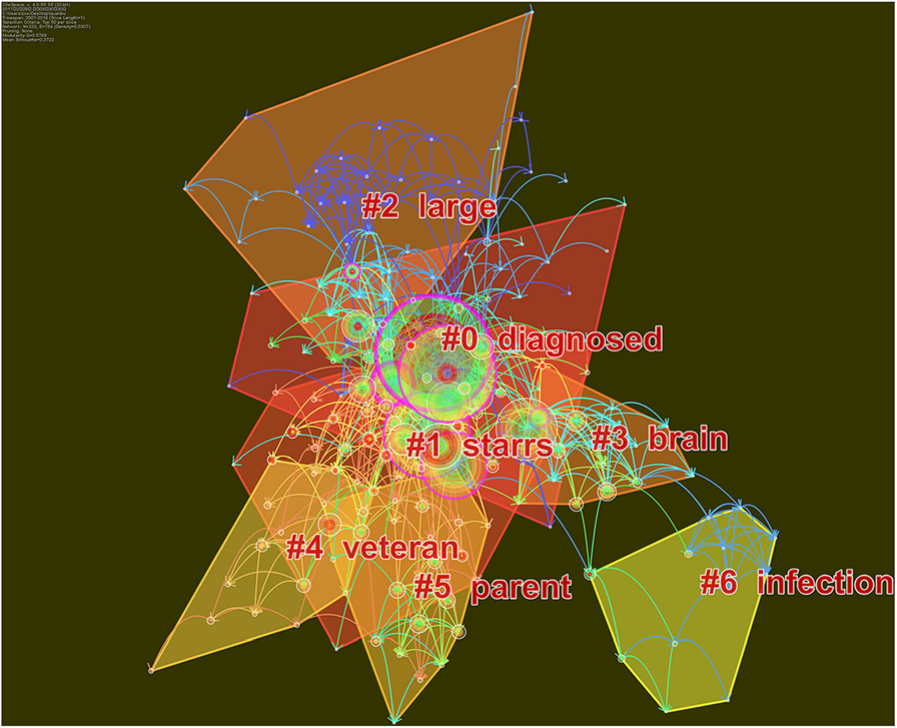
Reference co-citation network mapping of the articles related to military health and medicine research published from 2007 to 2016 #0:Mental health diagnoses and interventions; #1: Army study to assess risk and resilience in service members (STARRS); #2: Large-scale military action; #3: Brain science; #4: Veterans; #5: Soldier parents and children of the wartime; #6: Wound infection; The co-citation network mapping (222) and the number of connection lines (754) were obtained. The network modularity value is 0.5769, which is between 0 and 1. A greater value indicates better modularity of the clustering network. Meanwhile, the Silhouette value is 0.3722, which is between −1 and 1. A larger Silhouette value indicates a clearer theme for each cluster

**Fig. 2:**
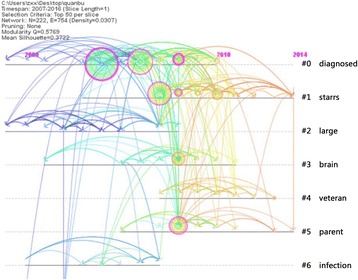
Reference co-citation time-views mapping of articles related to military health and medicine research published from 2007 to 2016. The co-citation network mapping (222) and the number of connection lines (754) were obtained. The network modularity value is 0.5769, which is between 0 and 1, and a greater value indicates better modularity of the clustering network. Meanwhile, the Silhouette value is 0.3722, which is between −1 and 1. A larger Silhouette value indicates a clearer theme for each cluster

In this study, co-citation network mapping (Fig. 1) and co-citation time-view mapping (Fig. 2) of the papers published in the WoSCC in the past 10 years have been conducted according to a comprehensive analysis of 7921 articles. The detailed process was as follows: First, in the function area and the parameter area of CiteSpace, the time zone division to 1 (that is, a one-year time slice) was set, and keywords and index terms such as the title, the abstract and the authors in the keyword extraction area were simultaneously selected and the reference co-citation was selected as the node type and Top 50 per slice as the threshold. Then, the CiteSpace III software was run and the number of nodes for the corresponding co-citation network mapping (222) and the number of connection lines (754) were obtained. From the parameter area at the upper left corner of the obtained network, we can see that the network modularity value is 0.5769. The modularity value is the evaluation index that reflects the degree of network modularization. The value of modularity is between 0 and 1 and a greater value indicates better modularity of the clustering network [8, 9]. Meanwhile, the Silhouette value is 0.3722. The Silhouette value is an index that measures network homogeneity, which is between −1 and 1. A larger Silhouette value indicates a clearer theme for each cluster [10–12].

### Research topics in military health and medicine

The above-mentioned 222 articles underwent a research hotspot cluster analysis. The top 7 clusters containing a total of 177 articles are listed Table 1. In the study, we used the Silhouette value to measure the homogeneity of the research topics and used the Log likelihood ratio (LLR) to analyze the current research topics. The Silhouette value of a cluster measures the quality of the clustering configuration. Its value ranges between −1 and 1. The highest value represents a perfect solution. LLR is a statistical test used for the comparison of the goodness of fit of two models.

**Table 1:** The co-citation of military health and medicine articles from 2007 to 2016

Ranking	Article counts		Silhouette value	Key words of research topics
	*n*	%		
0	45	25.4	0.711	Diagnosed; stress; health; Iraq
1	40	22.6	0.651	Army STARRS; health; veterans; mental
2	33	18.6	0.863	Large-scale military action; cohort study; Afghanistan war
3	17	9.6	0.905	Brain science; traumatic; injury
4	15	8.5	0.903	Veterans; women; sexual
5	15	8.5	0.829	Soldier parental; children; wartime
6	12	6.8	0.933	Wound infection; injuries; sustain; combat

*n*: Article counts; Silhouette value: a cluster measures the quality of a clustering configuration. Its value ranges between −1 and 1. The highest value represents a perfect solution; *STARRS*: study to assess risk and resilience in service members

Correlating the reference co-citation network mapping (Fig. 1) with Table 1, we can see that in the past 10 years, military health and medicine research has primarily focused on the following seven aspects obtained by LLR. #0: Mental health diagnoses and interventions accounted for 25.4% of all listed works, including a total of 45 papers, and had a Silhouette value of 0.711. The research contents included mental health assessments of naval crews who had been engaged in operations on naval vessels or other closed environments for a long period of time. #1: Army STARRS accounted for 22.6% of the articles, including a total of 40 papers, and had a Silhouette value of 0.651. These works included assessments of the health status of veterans and other related research. #2: Large-scale military action accounted for 18.6% of the papers, including 33 papers, and had a Silhouette value of 0.863. Using the war in Iraq as an example, the research involved social anxiety disorder and social fears in the USA military surgical treatment of war wounds and a summary of war wound diagnosis and treatment experiences. #3: Brain science accounted for 9.6% of the studies, with a total of 17 papers, and had a Silhouette value of 0.905. The research content involved the correlates of anger and hostility in Iraq and Afghanistan veterans and the mental health problems in these soldiers, including posttraumatic stress disorder (PTSD), brain injury, and alcohol abuse. #4: Veterans accounted for 8.5% of the studies, including a total of 15 articles, and had a Silhouette value of 0.903. The research content involved veterans’ poorer general health and greater incidence of health risk behaviors, mental health conditions, and chronic health conditions than civilian women. #5: Soldier parents and children of the wartime accounted for 8.5% of studies, with a total of 15 papers, and had a Silhouette value of 0.829. These studies included a research focus on family psychological health check-ins, family-specific psycho-education, family narrative timelines, and the family-level resilience skills of the military family members. #6: Wound infection accounted for 6.8% of papers, with a total of 12 articles, and had a Silhouette value of 0.933. These studies included the construction of a postoperative complication rate among American military members treated for fractures of the facial skeleton with either immediate fixation in the operation Iraqi and Afghanistan freedom combat theater or delayed fixation after transport out of the combat theater. As shown in the reference co-citation network mapping (Fig. 1), among these seven aspects of military health and medicine research in the past 10 years, a relatively close association can be observed between all aspects, with the exception of #5. In particular, the highest degree of network overlapping occurs between #0 and #1, indicating an even closer affiliation between these two. Furthermore, the correlation between each research field is further strengthened by the key nodes (those with purple outer circles).The results of the research for each aspect could provide a theoretical basis for another field of research so that the field of military medicine is divided into different research subfields [13]. In addition, the clusters in Fig. 1 are linked together by the bridge nodes (i.e., nodes at the junctions of the cluster groups), converging the various subfields of military medicine research into a unified entity that interactively penetrates, influences and promotes each subfield.

### High-yield authors and cooperative networks

**Fig. 3:**
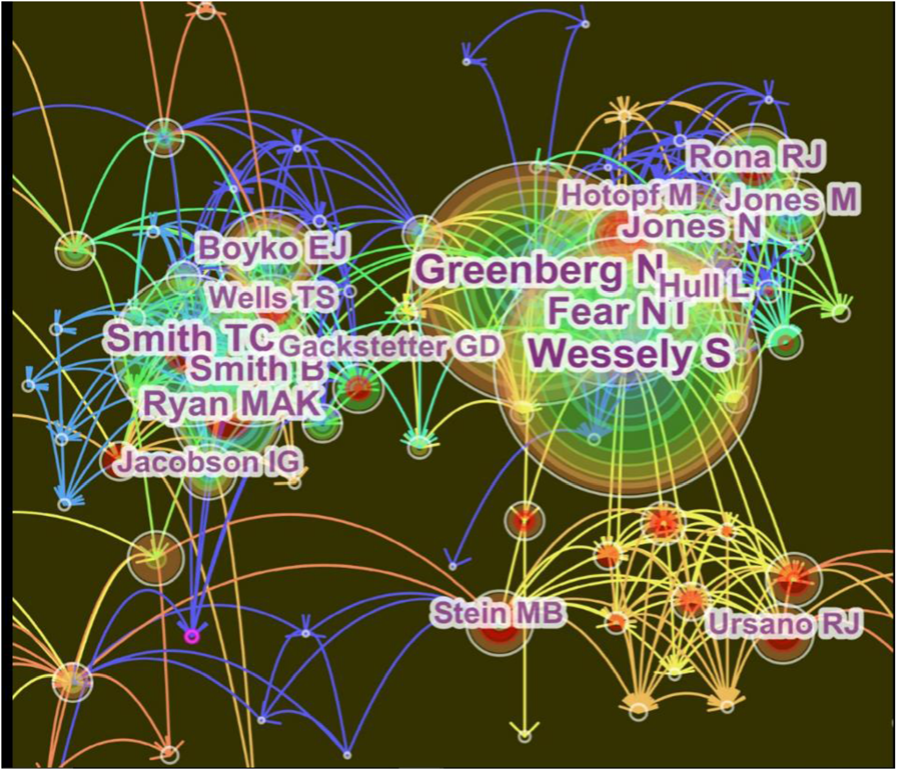
Mapping of high-yield authors and cooperative networks of articles related to military health and medicine published from 2007 to 2016. The top 10 high-yield researchers in military health and medicine research are Wessely S, Greenberg N, Fear NT, Smith TC, Smith B, Jones N, Ryan MAK, Boyko EJ, Hull L, and Rona RJ. The *nodes* in the map represent the corresponding authors. The *node size* represents the number of all document types of literature published by the author in the WoSCC. The *connection lines* between the nodes represent the cooperative relationships between the authors in the field and the thickness of the lines is positively correlated to the cooperation between the authors. The *color* of the *lines* represents the time of the first cooperation between the two authors

All of the data samples obtained from the WoSCC were imported into CiteSpace III software. Author was selected as the node type, the Threshold was set at Top 30 per slice, MST was chosen for Pruning, and the other settings were the same as those for the co-citation analysis. Then, CiteSpace III was run to obtain the cooperative network map for the authors whose articles were included in this study (Fig. 3). The nodes in the map represent the corresponding authors. The node size represents the number of all document types of literature published by the author in the WoSCC. The connection lines between the nodes represent the cooperative relationships between the authors in the field and the thickness of the lines is positively correlated to the cooperation between the authors. Moreover, the color of the lines represents the time of the first cooperation between the two authors.

As seen from Fig. 3, the top ten high-yield authors who have had the largest number of papers published during the last 10 years can be considered academic leaders or authoritative experts in the field of military health and medicine research. Meanwhile, core authors, as represented by a high output, tend to appear in the map in clusters and often maintain good cooperative relationships with other authors. For example, quite a few connection lines are observed between Wessely S and other scholar nodes, indicating that the author has a close relationship with other scholars in this field. As revealed by the software analysis results, the top 10 scholars with the largest number of papers published between 2007 and 2016 are as follows (Table 2): Wessely S of the Kings College London published 102 articles; Greenberg Nof the Kings College London published 92 articles; Fear NT of the Kings College London published 86 articles; Smith TC of the United States Navy published 69 articles; Smith B of the Naval Health Research Center published 58 articles; Jones N of the Kings College London published 50 articles; Ryan MAK of the Naval Hospital Camp Pendleton published 51 articles; Boyko EJ of the Puget Sound Healthcare Center published 43 articles; Hull L of the Kings College London published 45 articles; Rona RJ of the Kings College London published 41 articles.

**Table 2:** The top 10 high-yield authors with cited frequency in military health and medicine from 2007 to 2016

Ranking	Author	Institution	Cited frequency	Article count	Year
1	Wessely S	Kings College London	120	102	2007
2	Greenberg N	Kings College London	109	92	2007
3	Fear NT	Kings College London	94	86	2007
4	Smith TC	United States Navy	76	69	2007
5	Smith B	Naval Health Research Center	66	58	2007
6	Jones N	Kings College London	56	50	2010
7	Ryan MAK	Naval Hospital Camp Pendleton	55	51	2007
8	Boyko EJ	Puget Sound Healthcare Center	49	43	2009
9	Hull L	Kings College London	48	45	2007
10	Rona RJ	Kings College London	46	41	2007

As shown in Table 2, the top-ranked item by citation count is Wessely S in 2007, with a citation count of 120. The second one is Greenberg N in 2007, with a citation count of 109. The third is Fear NT in 2007, with a citation count of 94. The 4th is Smith TC in 2007, with a citation count of 76. The 5th is Smith B in 2007, with a citation count of 66. The 6th is Jones N in 2010, with a citation count of 56. The 7th is Ryan MAK in 2007, with a citation count of 55. The 8th is Boyko EJ in 2009, with a citation count of 49. The 9th is Hull L in 2007, with a citation count of 48. The 10th is Rona RJ in 2007, with a citation count of 46.

The betweenness centrality of a node in a network measures the extent to which the node is part of a path that connects an arbitrary pair of nodes in the network. As shown in Table 3, the top-ranked item by centrality is Hoge CW of the Walter Reed Army Institute of Research in 2007, with a centrality of 0.19. The second one is Bliese PD of the Walter Reed Army Institute of Research in 2011, with a centrality of 0.15. The third is Riddle JR of the United States Navy in 2007, with a centrality of 0.15. The 4th is Wells TS of the United States Navy in 2007, with a centrality of 0.13. The 5th is Riviere LA of the United States Navy in 2014, with a centrality of 0.13. The 6th is Wilk JE of the Walter Reed Army Institute of Research in 2010, with a centrality of 0.11. The 7th is Zamorski MA of the Canadian Forces Health Services Group in 2013, with a centrality of 0.10. The 8th is Castro CA of the University of Southern California in 2010, with a centrality of 0.10. The 9th is Sareen J of the University of Manitoba in 2010, with a centrality of 0.09. The 10th is Fear NT of the Kings College London in 2007, with a centrality of 0.08.

**Table 3:** The top 10 high-yield authors with cited Centrality in military health and medicine from 2007 to 2016

Ranking	Author	Institution	Centrality	Year
1	Hoge CW	Walter Reed Army Institute of Research	0.19	2007
2	Bliese PD	Walter Reed Army Institute of Research	0.15	2011
3	Riddle JR	United States Navy	0.15	2007
4	Wells TS	United States Navy	0.13	2007
5	Riviere LA	United States Army	0.13	2014
6	Wilk JE	Walter Reed Army Institute of Research	0.11	2010
7	Zamorski MA	Canadian Forces Health Services Group	0.10	2013
8	Castro CA	University of Southern California	0.10	2010
9	Sareen J	University of Manitoba	0.09	2010
10	Fear NT	Kings College London	0.08	2007

Centrality: a node in a network measures the extent to which the node is part of the path that connects an arbitrary pair of nodes in the network

**Fig. 4:**
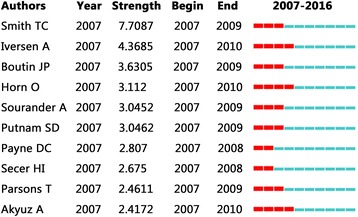
Top 10 authors with strongest citation bursts of military health and medicine research from 2007 to 2016. The top 10 ranked authors by bursts are Smith TC with bursts of 7.7087; Iversen A with bursts of 4.3685; Boutin JP with bursts of 3.6305; Horn O with bursts of 3.112; Sourander A with bursts of 3.0452; Putnam SD with bursts of 3.0452; Payne DC with bursts of 2.087; Secer HI with bursts of 2.675; Parsons T with bursts of 2.4611, and Akyuz A with bursts of 2.4172

Figure 4 shows that the top-ranked item by burst detection (The burst of the frequency of an entity over time indicates a specific duration in which an abrupt change of the frequency takes place. A citation burst has two attributes: the intensity of the burst and how long the burst status lasts.) is Smith TC in 2007, with bursts of 7.7087. The second one is Iversen A in 2007, with bursts of 4.3685. The third is Boutin JP in 2007, with bursts of 3.6305. The 4th is Horn O in 2007, with bursts of 3.112. The 5th is Sourander A in 2007, with bursts of 3.0452. The 6th is Putnam SD in 2007, with bursts of 3.0452. The 7th is Payne DC in 2007, with bursts of 2.087. The 8th is Secer HI in 2007, with bursts of 2.675. The 9th is Parsons T in 2007, with bursts of 2.4611. The 10th is Akyuz A in 2007, with bursts of 2.4172.

### Outstanding research institutions and cooperative networks

**Fig. 5:**
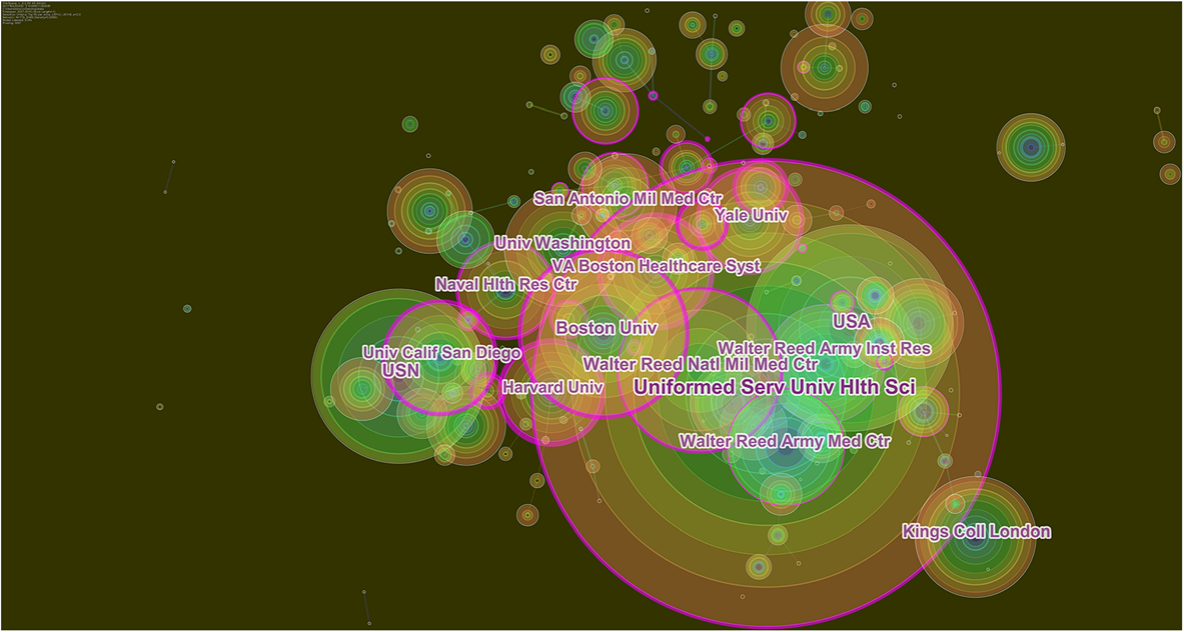
Mapping of the military health and medicine research institutions cooperative network from 2007 to 2016. The top 10 institutes were the Uniformed Services University of the Health Sciences, the United States Army, the United States Navy, Kings College London, Walter Reed National Military Medical Center, Boston University, Walter Reed Army Institute of Research, Walter Reed Army Medical Center, Naval Health Research Center, and VA Boston Healthcare System. The *size* of the *node ring* represents the number of papers published by this institution. The *nodes* with *purple outer rings* are institutions with high-level centrality and those with red inner rings are institutions with a high level of burst values

The quantity and quality of research output are objective indicators that reflect the research strength of scientific research institutions. In this study, the research strength of the institutions was explored and analyzed from the perspectives of the quantity and the quality of the literature. Throughout the research process, we used the number of publications as the basic evaluation index for military health and medicine research institutions. The centrality and burst value of the institutions were used as the evaluation criteria for the quality of the published articles and these values were used to analyze the distribution of military health and medicine research institutions with significant academic achievements and influence. Based on this, 7921 retrieved articles were imported into CiteSpace III, with the node type set as “Institution” and the other settings set to the same as in the co-citation analysis. Then, the mapping of cooperative networks of military health and medicine research institutions was achieved (Fig. 5).

As shown in Fig. 5, the size of the node ring represents the number of papers published by this institution. The nodes with purple outer rings are institutions with high-level centrality and those with red inner rings are institutions with a high level of burst values. Based on the analysis in Fig. 5, combined with the information table on network nodes that was automatically generated by CiteSpace III software (Tables 4, 5), the top ten research institutions with the largest number of publications are as follows: Uniformed Services University of the Health Sciences, the United States Army, the United States Navy, Kings College London, Walter Reed National Military Medical Center, Boston University, Walter Reed Army Institute of Research, Walter Reed Army Medical Center, Naval Health Research Center, and the VA Boston Healthcare System.

**Table 4:** The top 10 citation counts of military health and medicine research institutions from 2007 to 2016

Ranking	Citation counts	Institution	Year
1	427	Uniformed Services University of the Health Sciences	2007
2	220	United States Army	2007
3	193	United States Navy	2007
4	162	Kings College London	2007
5	152	Walter Reed National Military Medical Center	2012
6	132	Boston University	2007
7	118	Walter Reed Army Institute of Research	2007
8	112	Walter Reed Army Medical Center	2007
9	108	Naval Health Research Center	2007
10	105	VA Boston Healthcare System	2008

**Table 5:** Top 10 cooperation network nodes of military health and medicine research institutions from 2007 to 2016

Ranking	Centrality	Institution	year
1	0.39	Walter Reed Army Medical Center	2007
2	0.35	Uniformed Services University of the Health Sciences	2007
3	0.35	University of California Los Angeles	2010
4	0.34	Harvard University	2007
5	0.33	Madigan Army Medical Center	2012
6	0.29	Walter Reed Army Institute of Research	2007
7	0.26	United States Army	2007
8	0.18	Stanford University	2012
9	0.13	University of California San Diego	2007
10	0.12	San Antonio Military Medical Center	2011

As shown in Table 4, the top-ranked item by citation count is the Uniformed Services University of the Health Sciencesin 2007, with a citation count of 427. These citation counts rank first because they are significantly higher than those of other military health and medicine research institutions. Based on a query on the corresponding node of the Uniformed Services University of the Health Sciences, we can see that over the past 10 years, this university published more than 20 papers each year in the journal of *Military Medicine* and the number increased to 60 in 2015. Therefore, we believe that the Uniformed Services University of the Health Sciences is the front runner in military health and medicine research. The second is the United States Army in 2007, with a citation count of 220. The third is the United States Navy in 2007, with a citation count of 193. The 4th is the Kings College London in 2007, with a citation count of 162. The 5th is the Walter Reed National Military Medical Center in 2007, with a citation count of 152. The 6th is the Boston University in 2007, with a citation count of 132. The 7th is the Walter Reed Army Institute of Research in 2007, with a citation count of 118. The 8th is the Walter Reed Army Medical Center in 2007, with a citation count of 112. The 9th is the Naval Health Research Center in 2007, with a citation count of 108. The 10th is the VA Boston Healthcare System in 2008, with a citation count of 105 (Table 4).

As shown in Table 5, the top-ranked item by centrality is the Walter Reed Army Medical Center in 2007, with a centrality of 0.39. The second is the Uniformed Services University of the Health Sciences in 2007, with a centrality of 0.35. The third is the University of California Los Angeles in 2010, with a centrality of 0.35. The 4th is Harvard University in 2007, with a centrality of 0.34. The 5th is the Madigan Army Medical Center in 2007, with a centrality of 0.33. The 6th is the Walter Reed Army Institute of Research in 2007, with a centrality of 0.29. The 7th is the United States Army in 2007, with a centrality of 0.26. The 8th is Stanford University in 2012, with a centrality of 0.18. The 9th is the University of California San Diego in 2007, with a centrality of 0.13. The 10th is the San Antonio Military Medical Center in 2011, with a centrality of 0.12(Table 5).

In the analysis of the key nodes for network mapping, we can calculate the burst intensity of the network node by detecting the burst value, which is also one of the methods for determining the key nodes. After mapping the institution cooperative networks in CiteSpace III, based on citation counts, the institution with the greatest network (427) was the Uniformed Services University of the Health Sciences, indicating that it was the most active institution from 2007 to 2016 and played a pivotal role in the field of military health and medicine research. However, the institution with the highest centrality (0.39) was the Walter Reed Army Medical Center, which indicates that the Walter Reed Army Medical Center was central in institution cooperation in military health and medicine research.

### Regional analysis of the military health and medicine research

**Fig. 6:**
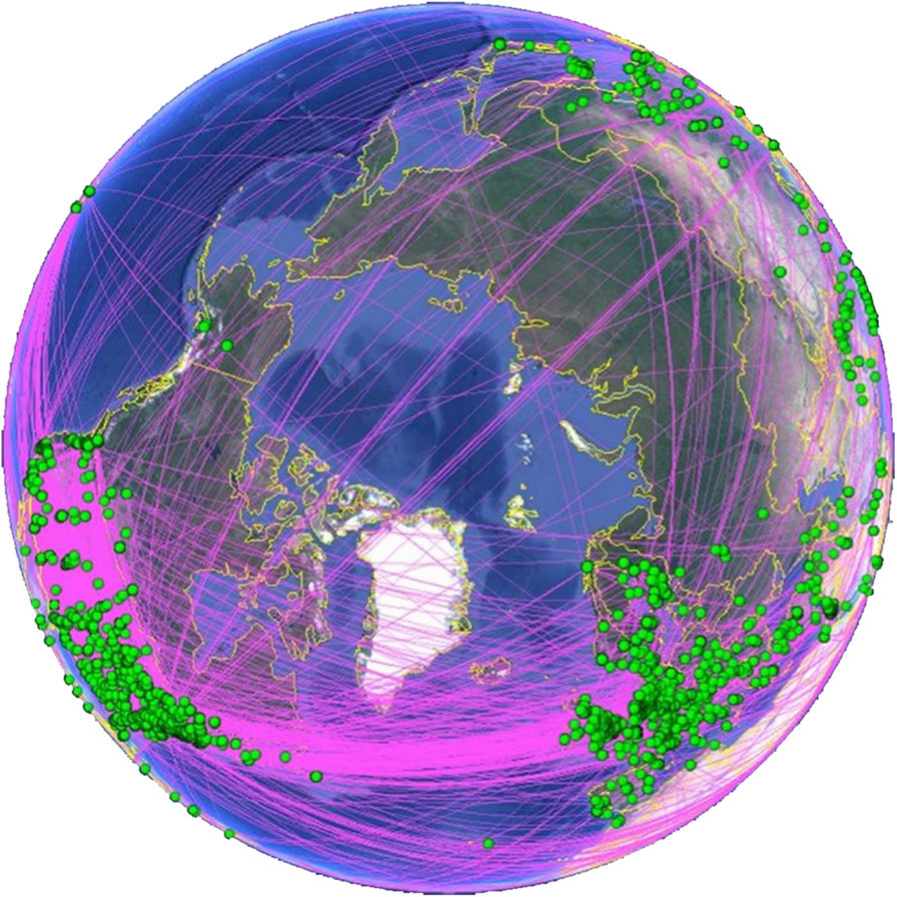
Regional analysis of the military health and medicine research from 2007 to 2016. Figure 6 shows that military health and medicine research is mainly distributed in Europe and the United States (*lower left* and *lower right* in Fig. 6, a *green point* represents a paper) and that other countries participate in less research

In this study, we used the geographic visualization function of CiteSpace to analyze the data. First, we selected the Geographical function and the KMZ file was obtained after setting the parameter and running CiteSpace. Then, we opened the KMZ file with Google Earth and generated the geographical distribution visualization map of military health and medicine research. Figure 6 shows an image of the military health and medicine research, demonstrating that the research is mainly contributed by Europe and the United States while other countries have less research. Through the above analysis of the distribution of military medical research, we believe that the European countries and the United States are still the leaders in military medical research.

## Discussion

In this study, we analyzed papers on military health and medicine published in the WoSCC from 2007 to 2016 using the text mining and visualization tool CiteSpace III and plotted the corresponding knowledge mapping to provide important reference information for follow-up research and management decisions in this field [14, 15]. The Silhouette value is used to measure the homogeneity of the articles co-citation network. In the articles co-citation network analysis, we used the Silhouette value to evaluate the reliability of clustering. The Silhouette values range is between −1 and 1and the closer the value is to 1, the higher the homogeneity of the articles co-citation network. When the Silhouette value is greater than 0.7, the clustering results have high reliability. When the Silhouette value is more than 0.5, the clustering results can be considered reasonable. As shown in Table 1, all Silhouette values are greater than 0.5 in this study, which shows that the clustering of articles co-citation is reasonable. After reviewing and analyzing the important articles and the relevant information in the mapping, we found that the research topics of military health and medicine in the WoSCC over the past 5 years mainly focused on the following seven aspects (#0 ~ #6). Based on the visualization analysis of the literature and a summary of the important articles, we believe that the current hot topics in military health and medicine research and the correlations between these top 3hot topics are as follows:

The largest cluster (#0) of the mental health diagnoses and intervention has 45 members and a Silhouette value of 0.711. This cluster group not only has the largest number of articles but also contains a number of key nodes in military health and medicine research. It is located in the center of military medicine research, with the highest level of centrality and the closest association with other clusters, indicating that mental health in war trauma is a long-term focus for military medicine research [16, 17]. It is labeled stress by the LLR and the most active citer to the cluster is Sundin, J (2010). PTSD after deployment to Iraq shows conflicting rates and conflicting claims. After reviewing the summary of reference co-citation clustering (Table 1) and the important studies with a high citation frequency and high centrality in this cluster knowledge group, we found that the research on war trauma and mental health mainly focuses on the assessment of mental health and psychological treatment following war trauma. The assessment method mainly involves an analysis of the psychological changes in personnel who have experienced major injuries or a poor combat environment based on the identification of evaluation factors in cohort studies, e.g., psychological evaluations of people who have worked in a closed environment for a long time, such as navy crews or drivers with challenging tasks and combatants who have been exposed to war [18].

The second largest cluster (#1) of the Army STARRS has 40 members and a Silhouette value of 0.651. It is labeled health by the LLR and the most active citer to the cluster is Jakupcak, M (2010). PTSD symptoms cluster in the relationship to alcohol misuse among Iraq and Afghanistan war veterans seeking post-deployment VA health care. This cluster group is very closely linked with Cluster #0 and a large number of nodes are connected by dense lines between the two clusters, indicating a large quantity of the same studies being cited by the research in these two clusters and reflecting the mapping relationship from the fundamental research to the frontier. Therefore, we can conclude that the studies from these two groups are complementary to each other and mutually reinforcing. As shown in Fig. 2, the literature cited by this cluster was largely concentrated in the period between 2007 and 2014, a fairly long time span, which means that research on post-deployment symptoms has been a key field in military health and medicine research in the past 10 years [19]. With the analysis of reference co-citation network mapping, the cluster for post-deployment symptoms was found to have several nodes that are closely related to Cluster #3, in addition to the strong link with Cluster #0 [2, 20].

The third largest cluster (#2) of large-scale military action has 33 members and a Silhouette value of 0.863. It is labeled large by both LLRs. The most active citer to the cluster is Wells, TS (2010), who provided a prospective study on depression following combat deployment in support of the wars in Iraq and Afghanistan. As shown in Fig. 1, this cluster is related to Cluster #0, indicating that medical rescue activities for those wounded in war are inextricably linked with basic military tactical training and cognitive function studies. It also shows that the subfields of military medicine research are closely correlated despite their different emphases [21]. Although orthopedic surgery research in Cluster #2 contains fewer nodes than Cluster #0, it has a close relationship with other cluster groups. Therefore, Cluster #2 has also been one of the most important aspects of military health and medicine research in recent years. Meanwhile, as can be found in Fig. 2, the literature cited by Cluster #2 research was mainly concentrated in the period from 2007 to 2011. By reading through the information in Table 1 and the key nodes contained in the Cluster #2 knowledge group, we found that the research for Cluster #2 involved social anxiety disorder and social fears regarding the military surgical treatment of war wounds and a summary of war wound diagnosis and treatment experiences [21–23].

## Conclusions

Visualization knowledge mapping was adopted in this study for a comprehensive analysis of military health and medicine research articles published in the WoSCC over the past 10 years. The WoSCC provides researchers, administrators, faculty, and students with quick, powerful access to the world’s leading citation databases. In this study, we summarized and analyzed current research topics in military health and medicine that mainly focus on the following seven aspects: mental health diagnoses and intervention, Army STARRS, large-scale military action, brain science, veterans, soldier parents and children of the wartime, and wound infection, respectively. The above research topics suggest that in future military health and medical research, we should give more attention to the medical support of war operations, posttraumatic stress disorder, the mental health of soldiers, the health protection of veterans, the health care of a soldiers’ family members, and the health of the servicemen. The top 10 high-yield researchers in military health and medicine research include Wessely S, Greenberg N, Fear NT, Smith TC, Smith B, Jones N, Ryan MAK, Boyko EJ, Hull L, and Rona RJ. Accordingly, the top 10 institutes were the Uniformed Services University of the Health Sciences, the United States Army, United States Navy, Kings College London, Walter Reed National Military Medical Center, Boston University, Walter Reed Army Institute of Research, Walter Reed Army Medical Center, Naval Health Research Center, and VA Boston Healthcare System. The above high-yield researchers and leading institutes were concentrated in Europe and the United States. This shows that a distance still exists between developing countries and European and American countries in military medical research. In addition, we will monitor the directions of current military health and medicine research and expand upon popular research fields by drawing on the experience of leading experts in this field. The research on the fundamental theory of military health and medicine should be emphasized and a theoretical research system should be established in this field. Moreover, enhancing military health and medicine practice and improving rescue capabilities for those wounded in war are necessary endeavors. In addition, military health and medicine education should be promoted to cultivate interdisciplinary military health and medicine rescue teams. The close cooperation between health care support and combat command departments should also be reinforced. Furthermore, we should actively encourage an international collaboration between outstanding research institutions in the field of military health and medicine to enhance the level of military health and medicine practice. Military health and medicine has faced new challenges and requirements with military transformation [1]. According to the European and the United States military health and medicine research, we need to expand the fields of the military health and medicine research, including natural disasters, military psychology, mental health, information on environmental medicine, aerospace medicine, military medical problems of the soldier system, war wound rehabilitation medicine, posttraumatic stress disorder, acute infectious diseases, and bioterrorism attacks. These topics will constitute the next developmental trend and research directions of military medical research.

Although our study included papers published on military health and medicine in the WoSCC over the past 10 years, understanding the general situation of military health and medicine research in recent years is possible. However, our study has several limitations. For example, the function of the software is limited and the retrieval strategy is incomplete. Our topic search terms could have underestimated the overall number of military health and medicine publications. Furthermore, the publication typing and tagging that is offered by the WoSCC may not be entirely accurate. From the recall ratio and the precision ratio of information retrieval, a stricter the retrieval condition is correlated with higher precision and the recall ratio becomes lower. Conversely, a broader retrieval condition is correlated with a higher recall rate and the precision ratio becomes lower. Therefore, any a search strategy is limited.

## Abbreviations

LLR: Log likelihood algorithm
PTSD: Posttraumatic stress disorder
STARRS: Study to assess risk and resilience in service members
WoSCC: Web of science™ core collection
